# Valorization of Beetroot and Watermelon by-Products as Functional Ingredients Targeting Nitric Oxide Pathways: Implications for Cardiovascular Health

**DOI:** 10.1007/s13668-026-00807-8

**Published:** 2026-10-17

**Authors:** Thiago Silveira Alvares

**Affiliations:** Food and Nutrition Institute, Multidisciplinary Center UFRJ-Macaé, Federal University of Rio de Janeiro, Estrada do Imburo, s/n, Macaé, Rio de Janeiro, 27979-000 Brazil

**Keywords:** Food waste valorization, Nitric oxide bioavailability, Functional foods, Cardiovascular health, Circular bioeconomy

## Abstract

**Purpose of the Review:**

Food loss and waste represent major challenges for food systems, while fruit and vegetable by-products generated during processing are underused sources of bioactive compounds with potential applications in functional food development. The present review aims to examine the valorization of beetroot and watermelon by-products as functional ingredients associated with nitric oxide (NO) bioavailability, vascular function, and cardiovascular health. Given that impaired NO signaling is a hallmark of endothelial dysfunction and cardiometabolic disease, nutritional strategies capable of modulating NO-related pathways are of growing interest.

**Recent Findings:**

Beetroot by-products, including peels and pomace, contain betalains, phenolic compounds, dietary fiber, and other constituents of potential functional relevance. However, nitrate content is rarely measured in these materials, and direct evidence that their consumption increases nitrate bioavailability or improves vascular function is currently lacking. Their proposed contribution to the nitrate–nitrite–NO pathway is therefore based mainly on evidence from conventional nitrate-rich beetroot products. In contrast, watermelon rind is a natural source of L-citrulline, which is bioavailable after ingestion and may support NO production through the citrulline–arginine–eNOS pathway. Human studies have demonstrated the bioavailability of L-citrulline derived from watermelon rind, while studies using a microencapsulated rind juice providing 4 g of L-citrulline have reported improvements in conduit artery endothelial function. Antioxidant compounds present in these by-products may also help limit oxidative degradation of NO, although this effect has not been directly demonstrated.

**Summary:**

Beetroot and watermelon by-products are linked to distinct pathways of NO generation but are currently supported by different levels of evidence. Future studies should quantify nitrate in beetroot by-products and L-citrulline in watermelon rind, including in the corresponding final food products, and evaluate changes in circulating nitrate, nitrite, and amino acids together with vascular function in well-controlled clinical studies.

**Abstract:**

**Figure:**
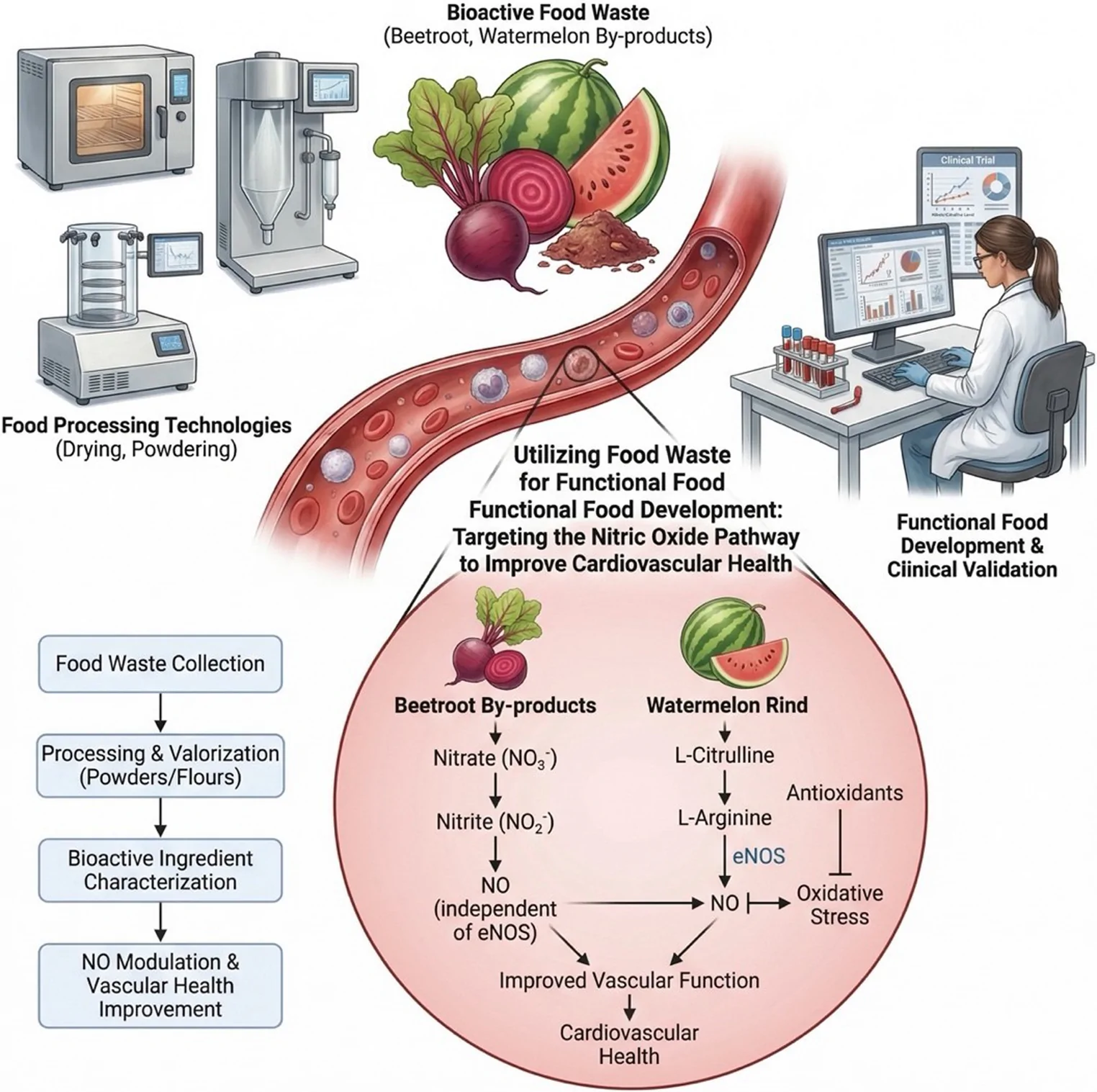

## Introduction

Food loss and waste represent a major challenge for contemporary food systems, generating substantial environmental burdens through greenhouse gas emissions, inefficient land and water use, and unnecessary energy expenditure across the supply chain [1–3]. Fruits and vegetables are among the most affected food categories, particularly due to their high perishability and the large volumes of by-products generated during industrial processing [1, 4, 5]. In recent years, food waste valorization has emerged as a central principle of the circular bioeconomy, shifting the focus from disposal toward the recovery of high-value compounds from underutilized biomass with potential applications in nutrition and health [1–3, 6].

Cardiovascular disease remains the leading cause of global morbidity and mortality, with endothelial dysfunction representing an early and central pathophysiological feature [7]. A defining characteristic of endothelial dysfunction is reduced nitric oxide (NO) bioavailability, which compromises vasodilation and promotes pro-atherogenic processes [7, 8]. Declines in NO signaling are consistently observed with aging and in cardiometabolic conditions such as obesity, hypertension, and type 2 diabetes, positioning NO as a key mechanistic target for dietary interventions aimed at preserving vascular health [7, 8]. NO bioavailability is regulated through multiple interconnected pathways that can be modulated by diet [7–10]. Endothelial NO synthase (eNOS)–dependent production relies on adequate substrate availability and favorable redox conditions, while the nitrate–nitrite–NO pathway provides an alternative, oxygen-independent source of NO that becomes particularly relevant when endothelial function is impaired [8, 11]. Nutrients such as inorganic nitrate and L-citrulline therefore represent promising targets for functional food development.

**Fig. 1:**
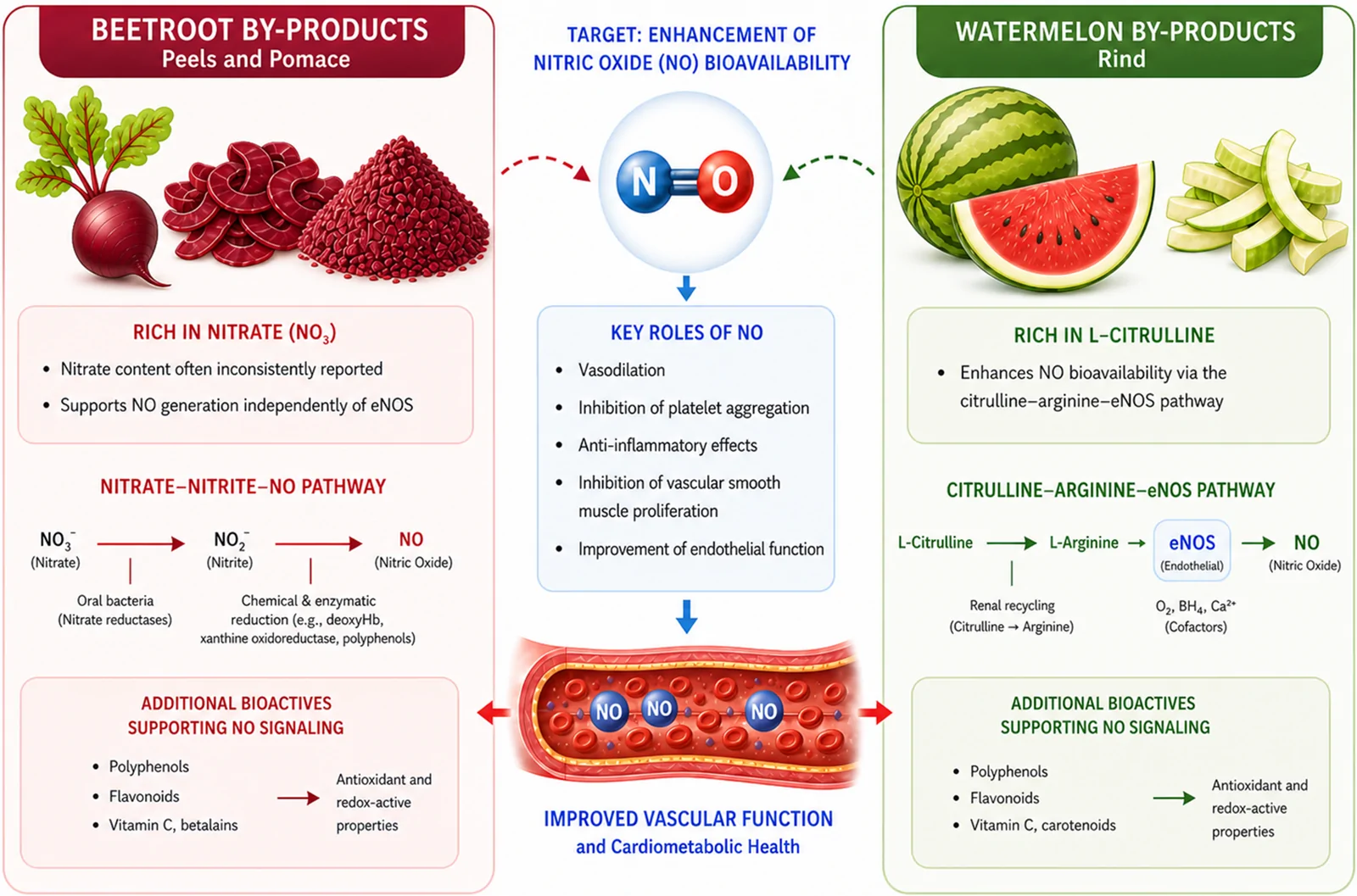
Valorization of fruit and vegetable by-products as complementary nutritional strategies to modulate nitric oxide bioavailability. Food loss and waste from fruit and vegetable processing generate substantial environmental burdens but also represent underutilized sources of bioactive compounds. Beetroot by-products, including pomace and peel, contain betalains, phenolic compounds, and other antioxidant constituents, whereas their nitrate content has rarely been reported. Their proposed contribution to the nitrate–nitrite–NO pathway is based on the established physiology of dietary nitrate and evidence from conventional beetroot products rather than direct studies demonstrating NO-related or vascular effects following consumption of beetroot peel or pomace. In contrast, watermelon rind–derived ingredients are rich in L-citrulline and may target NO bioavailability through the citrulline–arginine–eNOS pathway. Together, these distinct food wastes exemplify complementary nutritional approaches for supporting NO signaling while aligning cardiovascular health with sustainable food system valorization

Among the various fruit and vegetable by-products, beetroot and watermelon by-products were selected to examine two pathways relevant to NO bioavailability: the nitrate–nitrite–NO pathway and the citrulline–arginine–eNOS pathway. Beetroot peel and pomace have been proposed as sources of nitrate, although nitrate content has rarely been measured in these by-products [12–19]. In contrast, watermelon rind is a documented source of L-citrulline [20–23], which may increase L-arginine availability for eNOS-dependent NO synthesis [9, 24, 25]. The two by-products also differ in the type and strength of available evidence. Research on beetroot by-products has focused mainly on chemical composition, processing, stability, and incorporation into food products [12–17, 19, 26], with limited direct evaluation of NO-related or vascular outcomes. In contrast, watermelon rind has been evaluated in a small number of human studies assessing circulating amino acids and vascular function [21, 23, 27, 28]. Therefore, this review focuses on these two by-products to critically compare their proposed mechanisms, available evidence, limitations, and potential for development as functional ingredients targeting NO bioavailability. The proposed links between these by-products, their respective NO-generating pathways, and vascular function are summarized in Fig. 1.

Relevant studies were identified through searches of PubMed, Scopus, and Web of Science using combinations of terms related to food waste and by-products, beetroot, watermelon rind, nitrate, L-citrulline, nitric oxide, vascular function, processing, and functional food development. Reference lists of relevant original studies and reviews were also examined. Priority was given to studies directly evaluating beetroot or watermelon by-products, while studies involving conventional beetroot products, watermelon, dietary nitrate, or L-citrulline were used to support mechanistic interpretation.

## Food Waste and the Environment 

Food Waste and the EnvironmentApproximately one-third of the food produced for human consumption is lost or wasted along the food supply chain [2, 29]. These losses contribute to greenhouse gas emissions and the unnecessary use of land, freshwater, and energy [2, 3, 29]. Fruits and vegetables are particularly affected because of their high perishability and susceptibility to damage during harvesting, processing, distribution, and storage [1, 30]. In some regions, losses may exceed 40% of total fruit and vegetable production [1, 30].

Food waste valorization seeks to convert by-products and residues into products with greater value rather than directing them to disposal or lower-value uses [1, 5, 6]. Fruit and vegetable by-products may contain phytochemicals, amino acids, dietary fiber, and other compounds of nutritional interest [31]. In the context of this review, watermelon rind is relevant because it is a documented source of L-citrulline [21–23]. Beetroot peel and pomace have been investigated as sources of betalains, phenolic compounds, and dietary fiber, but their nitrate content remains poorly documented [12–19, 32].

The use of these by-products as food ingredients may provide an alternative to disposal and increase the value obtained from the original crops. However, environmental benefits cannot be assumed solely from the use of a by-product because processing, transportation, packaging, and storage also require resources. Comparative environmental assessments are therefore needed to determine whether their conversion into food ingredients offers advantages over their current use or management.

## Cardiovascular Function, Nitric Oxide, and Nutritional Modulation

Endothelial function is a central determinant of cardiovascular health and one of the earliest physiological processes impaired in cardiometabolic disease [7, 8, 33]. A main feature of endothelial dysfunction is reduced NO bioavailability, which compromises vasodilation and promotes platelet aggregation, inflammation, and vascular smooth muscle proliferation [7, 8]. Impaired NO signaling has been observed with aging and in conditions such as hypertension, obesity, and type 2 diabetes, positioning NO as a key mechanistic target for nutritional strategies aimed at promoting cardiovascular health [8, 33, 34].

Under physiological conditions, NO synthesis is primarily mediated by eNOS, which converts L-arginine into NO in response to shear stress [7, 9]. However, this pathway becomes progressively impaired in states of oxidative stress and metabolic dysfunction [8, 33, 34]. Reduced L-arginine availability and endogenous inhibitors such as asymmetric dimethylarginine (ADMA) decrease eNOS activity, whereas oxidation of eNOS cofactors (e.g., tetrahydrobiopterin – BH4) leads to eNOS uncoupling, superoxide generation, and diminished NO bioavailability [8, 9]. These limitations help explain why strategies exclusively targeting endogenous eNOS activity may be insufficient to fully restore NO bioavailability in populations at elevated cardiovascular risk [8].

Beyond the classical eNOS-dependent pathway, NO bioavailability can be supported by the nitrate–nitrite–NO pathway, which operates independently of eNOS activity [8, 11]. Dietary nitrate is reduced to nitrite through the entero-salivary circulation and further converted to NO in blood and tissues, particularly under hypoxic and acidic conditions [11]. This pathway is of particular physiological relevance in settings where endothelial function is compromised, as it may provide an alternative source of NO when eNOS-dependent synthesis is limited. Therefore, nitrate-rich foods may represent a complementary nutritional strategy to sustain NO signaling in both health and disease. The effectiveness of dietary nitrate interventions is not determined solely by the amount of nitrate ingested. The initial reduction of salivary nitrate to nitrite depends largely on nitrate-reducing bacteria in the oral cavity [11, 35–37]. Antibacterial mouthwash has been shown to suppress oral nitrate reduction and decrease circulating nitrite concentrations [35, 36], while differences in the composition and activity of the oral microbiota may contribute to variability in NO homeostasis and vascular responses following nitrate intake [37]. Therefore, oral microbiota composition represents an important source of inter-individual variability when evaluating nitrate-rich foods and functional ingredients.

In parallel, the citrulline–arginine–eNOS pathway offers an additional nutritional mechanism to support NO production. Unlike L-arginine, L-citrulline escapes extensive intestinal and hepatic metabolism and is efficiently converted to L-arginine in the kidneys, leading to sustained increases in plasma L-arginine availability for eNOS-dependent NO synthesis [9]. This pathway has gained increasing attention because it bypasses key metabolic bottlenecks associated with direct L-arginine supplementation, particularly under conditions characterized by increased arginase activity and oxidative stress [9, 24, 25]. Although L-citrulline intake generally increases circulating L-citrulline and L-arginine concentrations [21, 25], this pharmacokinetic response does not necessarily result in a consistent improvement in vascular function [23, 28]. The effect on NO bioavailability may also depend on arginase activity, endogenous eNOS inhibitors such as ADMA, and the prevailing vascular redox environment [8, 9, 24, 25, 33, 34]. Therefore, increases in circulating L-citrulline and L-arginine should not be interpreted as evidence of improved vascular function.

The nitrate–nitrite–NO and citrulline–arginine–eNOS pathways differ in both the source of NO and the factors limiting their responses. The nitrate–nitrite–NO pathway depends on enterosalivary circulation and the reduction of nitrate to nitrite by oral bacteria, whereas the subsequent conversion of nitrite to NO does not require eNOS and is favored under hypoxic and acidic conditions [11, 35–37]. Consequently, responses to dietary nitrate may be influenced by oral microbiota composition and antibacterial mouthwash use. In contrast, L-citrulline acts indirectly by increasing L-arginine availability for eNOS-dependent NO synthesis [9, 24, 25]. Its vascular effects may therefore depend on endothelial eNOS function, L-arginine availability, arginase activity, endogenous eNOS inhibitors, and the prevailing vascular redox state [8, 9, 24, 25, 33, 34]. Oxidative stress can limit the effects of both pathways by accelerating NO degradation and increasing peroxynitrite formation [33, 34]. Differences in dose, food matrix, and cardiometabolic status may further contribute to variation in responses among individuals.

## Beetroot by-Products as Functional Ingredients Targeting Nitric Oxide Bioavailability

Beetroot (*Beta vulgaris L.*) processing generates by-products, including peels, pomace, and residual pulp, which are commonly discarded [14–16]. These by-products originate mainly from juice extraction and pigment production [17, 26]. During beetroot juice production, pomace may account for approximately 15–30% of the processed beetroot mass [38]. This proportion indicates that juice production generates a considerable amount of residual biomass. Studies of beetroot peels and pomace have primarily characterized betalains, phenolic compounds, dietary fiber, and antioxidant activity, as well as their suitability for incorporation into food products [12–17, 19, 32]. In contrast, nitrate concentration has rarely been measured in these by-products. Consequently, their capacity to supply nitrate and influence the nitrate–nitrite–NO pathway has not yet been demonstrated through assessments of nitrate bioavailability, NO-related biomarkers, endothelial function, or cardiovascular outcomes. Table 1 summarizes the selected studies investigating beetroot by-products.

**Table 1:** Selected studies evaluating beetroot and watermelon by-products as functional ingredients and their relevance to nitric oxide bioavailability and vascular function

Study	Study design/ population	By-product	Product transformed	Main nutrients/bioactives	Main findings
Šaponjac et al., [15]	Non-clinical In vitro analysis	Beetroot pomace	Encapsulated beetroot pomace extract	Polyphenols, flavonoids, and betalains; nitrate not reported	↑ antioxidant stability and bioaccessibility
Hidalgo et al., [13]	Non-clinical In vitro analysis	Beetroot pomace	Extracts/microencapsulates added to water biscuits	Betanin, isobetanin, and polyphenols; nitrate not reported	↑ antioxidant activity
Petrović et al., [26]	Non-clinical Chemical composition	Beetroot pomace	Apple/beetroot pomace liqueur	Phenolic compounds and betalains; nitrate not reported	↑ phenolic content and antioxidant activity
Gorjanović et al., [17]	Non-clinical and clinical Healthy adults	Beetroot pomace	Jelly candies	Dietary fiber, polyphenols, flavonoids, betacyanins, and betaxanthins; nitrate not reported	↓ postprandial glucose, glycemic index and glycemic load ↑ phenolic content and antioxidant activity
Hernández-Montesinos et al., [32]	Non-clinical In vitro analysis	Beetroot pomace	Ethanolic extract	Phenolic compounds and flavonoids; nitrate not reported	↑ phenolic and flavonoids content and antioxidant activity
Coimbra et al., [14]	Non-clinical In vitro analysis	Beetroot peel	Beetroot peel flour	Fiber, betacyanins, and betaxanthins; nitrate not reported	Chemoprotective and anticancer activity (in vitro)
Buterchi et al., [12]	Non-clinical In vitro analysis	Beetroot peel	Dehydrated apple snacks enriched with beetroot peel powder	Polyphenols and betalains; nitrate not reported	↑ phenolic content and antioxidant activity
Tarazona-Díaz et al., [22]	Non-clinical Chemical composition	Watermelon rind	Watermelon rind	L-citrulline (2.0 to 7.2 mg.g^− 1^ fresh weight), phenolics, antioxidants	L-citrulline content varied among cultivars
Fan et al., [23]	Clinical Overweight/ obese adults	Watermelon rind, flesh, and seeds	Meal preparations containing: fresh diced watermelon flesh, fresh watermelon rind, or dried watermelon seeds	L-citrulline (387 mg), L-arginine (75 mg), and polyphenols Single dose	↑ plasma L-citrulline and phenolic metabolites ↔ endothelial function
Volino-Souza et al., [27]	Clinical Healthy adults	Watermelon rind	Microencapsulated watermelon rind	L-citrulline (4 g) Single dose	↑ plasma L-citrulline and L-arginine ↑ endothelial function ↔ microvascular responsiveness
Volino-Souza et al., [21]	Clinical Healthy adults	Watermelon rind and pulp	Microencapsulated watermelon rind and pulp juices	L-citrulline (4 g) and L-arginine Single dose	↑ plasma L-citrulline and L-arginine Similar glycemic response to pulp
Volino-Souza et al., [28]	Clinical Healthy adults	Watermelon rind + beetroot	Microencapsulated watermelon rind juice alone or combined with microencapsulated beetroot juice	L-citrulline (4 g), nitrate (8 mmol) Single dose	↑ endothelial function ↔ microvascular responsiveness

↑ or ↓ indicate significant increases and decreases, respectively. ↔ indicates no significant change. Nitrate was not reported in the studies evaluating beetroot peel or pomace, and these studies did not directly assess nitrate bioavailability, NO-related biomarkers, or vascular function. Antioxidant, compositional, and glycemic findings were not considered direct NO-related or vascular outcomes.

Dietary nitrate is the principal substrate for NO generation through the nitrate–nitrite–NO pathway, and its physiological effects depend on the amount consumed, bioavailability, and food matrix [10, 11]. Although studies specifically measuring nitrate in beetroot peel and pomace remain scarce, studies using other beetroot products illustrate how processing and formulation may influence nitrate content. For example, Vasconcellos et al. [18] reported nitrate concentrations of approximately 12,250 mg·kg⁻¹ dry weight in beetroot juice, compared with 2,030 mg·kg⁻¹ in beetroot chips, 1,680 mg·kg⁻¹ in beetroot powder, and 1,650 mg·kg⁻¹ in cooked beetroot. These products were not specifically derived from peel or pomace. Therefore, these findings illustrate differences in nitrate content among processed beetroot products but do not provide direct information on nitrate concentration in beetroot by-products.

Nitrate concentration in beetroot may also differ before processing. Yasaminshirazi et al. [39] investigated 15 beetroot genotypes grown at three locations over two years and observed that nitrate concentrations ranged from approximately 4,593 to 10,924 mg·kg⁻¹ dry weight. Furthermore, the authors observed that the nitrate content of the investigated beetroots differed according to genotype, growing location, and year. Harvest time may also influence nitrate concentration. Takács-Hájos and Vargas-Rubóczki [40] evaluated five beetroot cultivars at different stages of growth and found higher nitrate concentrations in roots harvested after 105 days than in those harvested after 85 days. In addition, Yasaminshirazi et al. [41] investigated 36 beetroot genotypes and showed that nitrate concentration changed during cold storage, with the main changes occurring during the first month. These studies evaluated whole beetroot roots rather than peel or pomace; therefore, their nitrate values cannot be applied directly to beetroot by-products. Nevertheless, they indicate that cultivar, growing location and year, harvest time, and storage before processing should be reported when nitrate is measured in beetroot peel- or pomace-derived ingredients.

Most studies investigating beetroot peel and pomace have focused on phenolic compounds, betalains, antioxidant capacity, processing, or incorporation into food products without reporting nitrate concentration [12–17, 19, 32]. Consequently, the nitrate amount provided per gram of ingredient or per serving of the final product cannot be calculated. This information is essential because human studies reporting vascular effects with conventional beetroot products have used known and reported nitrate doses [10, 42–44]. However, these findings cannot be assumed to apply to beetroot peel or pomace. Until studies quantify nitrate in beetroot peel- and pomace-derived ingredients, assess changes during processing and storage, and report the amount provided per serving, it remains unknown whether these by-products can provide sufficient nitrate to influence the nitrate–nitrite–NO pathway.

Beetroot by-products contain betalains and phenolic compounds and have shown high antioxidant capacity in vitro [15, 32]. These characteristics may be relevant because oxidative stress accelerates NO degradation and limits its vascular actions [33, 34]. If beetroot by-products provide an amount of nitrate sufficient to influence NO production, the antioxidant compounds present in the same matrix (e.g., betalains and phenolic compounds) could potentially help limit oxidative NO degradation. Moreover, antioxidant capacity demonstrated in in vitro studies should not be interpreted as evidence that consumption of these by-products increases NO bioavailability or improves vascular function in humans. Beetroot peel and pomace can be dried and transformed into powders or flours for incorporation into snacks, bakery products, crispbreads, candies, and other food formulations [12, 16, 17, 19]. Their incorporation may increase dietary fiber, betalain and phenolic content, antioxidant capacity, and natural coloration, although the effects depend on the amount added and the processing conditions [12, 13, 16, 17, 19]. These studies demonstrate the feasibility of using beetroot by-products in food products but do not establish effects on nitrate bioavailability or vascular function.

Encapsulation of beetroot pomace extracts has been investigated as a strategy to improve the stability and bioaccessibility of betalains and phenolic compounds [13, 15]. A study from our group using microencapsulated beetroot juice reported that nitrate remained stable over 32 days at all storage temperatures evaluated; however, the product was produced from beetroot juice rather than peel or pomace [45]. Consequently, these findings on nitrate stability cannot be extrapolated directly to beetroot by-products. Future studies should measure nitrate concentrations before and after drying, encapsulation, and storage and report the nitrate amount provided per serving of the final product.

Overall, beetroot peel and pomace have demonstrated value as sources of betalains, phenolic compounds, and dietary fiber and as ingredients for incorporation into different food products. However, their capacity to increase NO bioavailability has not been demonstrated. The proposed link with the nitrate–nitrite–NO pathway is currently based on the established physiology of dietary nitrate and on studies using conventional beetroot products with measured nitrate content. Future studies should quantify nitrate in both the by-product ingredient and the final food product, determine the amount provided per serving, assess changes in circulating nitrate and nitrite after consumption, and evaluate effects on endothelial function, blood pressure, and other cardiovascular outcomes.

## Watermelon by-Products as Functional Ingredients Targeting Nitric Oxide Bioavailability

Watermelon (*Citrullus lanatus*) processing and consumption generate rind and seeds as the main by-products. The rind represents approximately 40% of the total fruit weight [46], indicating that a considerable fraction of the fruit is not included in the commonly consumed pulp. Global watermelon production reached approximately 118 million tons in 2017 [46], suggesting that the potential amount of rind available for valorization is significant. However, this estimate should not be interpreted as the amount actually discarded because part of the rind may be used as animal feed, incorporated into food products, or managed in other ways. Watermelon rind has attracted interest as a food ingredient because it contains higher concentrations of L-citrulline than the pulp [22, 23]. Its utilization may therefore provide an opportunity to convert an underused part of the fruit into an ingredient with potential applications related to NO bioavailability [20].

L-citrulline concentration in watermelon rind is not consistent among fruits. Tarazona-Díaz et al. [22] reported L-citrulline concentrations in watermelon rind ranging from approximately 2.0 to 7.2 mg·g^− 1^ fresh weight among five watermelon cultivars. In a study investigating 56 watermelon cultigens, including cultivars, breeding lines, and accessions [47], L-citrulline concentration in watermelon fruit ranged from approximately 1.09 to 4.52 mg.g^− 1^ fresh weight. The mean concentration was also higher in fruit grown in Oklahoma than in fruit grown in Texas (3.10 versus 1.67 mg.g^− 1^ fresh weight), demonstrating effects of both genotype and growing environment. Because the latter study assessed watermelon fruit rather than rind-derived ingredients, the reported L-citrulline concentrations should not be extrapolated to watermelon rind products. Together, these findings indicate that cultivar and growing location should be reported and that L-citrulline concentration should be measured in the rind used to prepare each ingredient rather than inferred from values reported for other cultivars or production regions. L-citrulline should also be measured after processing and during storage of the final ingredient.

Unlike beetroot by-products, whose NO-enhancing potential is primarily mediated through dietary nitrate, watermelon rind products may target NO bioavailability via the citrulline–arginine–eNOS pathway [20, 23, 24]. L-citrulline is efficiently absorbed and largely escapes first-pass intestinal and hepatic metabolism, being converted to L-arginine in the kidneys and thereby sustaining substrate availability for eNOS–dependent NO production [24, 25]. This pathway bypasses important limitations associated with L-arginine supplementation, including its substantial metabolism by intestinal and hepatic arginase before reaching the systemic circulation [9, 24, 25]. In addition to its L-citrulline content, watermelon rind–derived products may differ from pulp in their sugar composition. In a study from our group, Volino-Souza et al. [21] compared microencapsulated watermelon rind and pulp juices, each providing 4 g of L-citrulline, in healthy adults. The microencapsulated watermelon rind juice contained lower concentrations of sucrose, glucose, and fructose than the microencapsulated watermelon pulp juice, but the two products produced similar postprandial glycemic responses. Because the study was conducted in healthy adults, these findings do not indicate whether the lower sugar content of the rind formulation would influence glycemic responses in individuals with obesity, insulin resistance, or type 2 diabetes. This possibility should therefore be evaluated further in populations with impaired glucose regulation.

Human studies specifically evaluating watermelon rind remain limited. One study from our group examined the bioavailability of L-citrulline from a microencapsulated watermelon rind juice [21]. Eleven healthy adults ingested a single dose of microencapsulated watermelon rind juice or microencapsulated watermelon pulp juice, with both products providing 4 g of L-citrulline. Plasma L-citrulline, L-arginine, and L-ornithine concentrations were assessed before ingestion and during the subsequent 120 min. Both products increased plasma L-citrulline concentrations, whereas watermelon rind produced an earlier increase in plasma L-arginine. These findings demonstrated that L-citrulline from microencapsulated watermelon rind juice was bioavailable. In another study from our group [28], participants ingested a single dose of microencapsulated watermelon rind juice providing 4 g of L-citrulline or placebo. Plasma amino acids and brachial artery flow-mediated dilation (FMD), a measurement of endothelial function, were assessed before and at 60 and 120 min after intervention. Microvascular responsiveness, evaluated by changes in tissue oxygen saturation during the reperfusion phase of a vascular occlusion test using near-infrared spectroscopy, was assessed before ingestion and at 30, 60, 90, and 120 min. Watermelon rind increased plasma L-citrulline and L-arginine concentrations and improved FMD at 60 min, whereas no significant effects were observed on microvascular responsiveness. Fan et al. [23] evaluated six adults with overweight or obesity who consumed meals containing watermelon rind, flesh, or seeds, or a control meal. The fresh watermelon rind serving provided approximately 387 mg of L-citrulline and 75 mg of L-arginine. Plasma amino acids and brachial artery FMD were assessed during the 7-h postprandial period. Watermelon rind increased circulating L-citrulline and L-arginine concentrations but did not improve FMD. Therefore, this study also demonstrated the bioavailability of amino acids from watermelon rind but did not show an acute effect on endothelial function. The lower L-citrulline dose may have contributed to the absence of an FMD response, because the serving provided approximately 0.4 g of L-citrulline, compared with 4 g in the studies using microencapsulated watermelon rind juice [27, 28]. Nevertheless, the studies also differed in participant characteristics, product formulation, meal composition, and experimental protocol, preventing the different FMD responses from being attributed to dose alone. Another randomized, placebo-controlled crossover study evaluated whether microencapsulated watermelon rind juice could attenuate endothelial dysfunction induced by prolonged arterial occlusion [27]. Healthy adults ingested a single dose of watermelon rind providing 4 g of L-citrulline, watermelon rind combined with microencapsulated beetroot juice, or placebo. FMD and microvascular responsiveness were assessed before and after 20 min of forearm ischemia. Microencapsulated watermelon rind juice alone and in combination with beetroot attenuated the reduction in FMD induced by prolonged ischemia. However, no protective effect was observed on microvascular responsiveness. Furthermore, microencapsulated beetroot juice co-ingestion did not provide an additional benefit compared with microencapsulated watermelon rind juice alone. Collectively, these studies show that L-citrulline from watermelon rind is bioavailable and suggest that a single dose of a microencapsulated watermelon rind juice providing 4 g of L-citrulline may acutely improve conduit artery endothelial function. The different FMD responses reported by Fan et al. [23] and in the studies from our group suggest that L-citrulline dose and product formulation may influence the vascular response, although no direct dose–response study has been conducted. The available evidence remains limited by small sample sizes, acute interventions, and the predominance of healthy adults. More studies using comparable watermelon rind formulations, longer supplementation periods, and populations with cardiometabolic risk are therefore needed. Selected studies evaluating watermelon by-products are summarized in Table 1.

Overall, watermelon rind is a documented source of L-citrulline [20–23, 27, 28, 45], and the available human studies support the bioavailability of rind-derived L-citrulline [21, 23]. Single-dose studies using microencapsulated watermelon rind juice have also reported improvements in conduit artery endothelial function [27, 28]. These findings provide preliminary human evidence for the use of watermelon rind as a functional ingredient targeting the citrulline–arginine–eNOS pathway.

## Conclusions and Future Perspectives in Food Waste Utilization

Food waste valorization represents an opportunity to develop functional ingredients from underused fruit and vegetable materials. Beetroot and watermelon by-products are linked to distinct NO-related pathways, but the type and strength of supporting evidence differ substantially. Studies of beetroot peel and pomace have mainly evaluated phenolic compounds, betalains, dietary fiber, antioxidant activity, and incorporation into food products [12–17, 19, 26, 32]. Because nitrate content and vascular outcomes have rarely been evaluated, the capacity of beetroot peel and pomace to influence the nitrate–nitrite–NO pathway remains to be demonstrated. In contrast, watermelon rind is a documented source of L-citrulline [22, 23] and has been evaluated in small acute human studies assessing amino-acid bioavailability and vascular function [21, 23, 27, 28].

Future research should address three main priorities. First, future research should move beyond antioxidant capacity and compositional analyses toward dose–response studies linked to validated vascular endpoints, including endothelial function and NO-related biomarkers. Second, studies focused on food formulation and processing are needed to preserve nitrate, L-citrulline, and other relevant compounds during processing and storage and to ensure a consistent amount of these compounds in each serving of the final product. Beetroot and watermelon are commonly consumed foods, and the available human studies using watermelon rind and beetroot-containing formulations did not report relevant adverse effects at the doses evaluated [21, 27, 28]. Nevertheless, the production of ingredients from beetroot peel, beetroot pomace, or watermelon rind requires the routine quality controls applied to processed foods, including documentation of the origin and handling of the raw material, appropriate washing and sanitation procedures, microbiological assessment, evaluation of potential contaminants, and suitable packaging and storage conditions [48–50]. Studies should also report the cultivar, growing location and year, harvest stage, and storage conditions before processing, together with the nitrate or L-citrulline content measured in the ingredient used in each study. Future studies should also determine whether these ingredients can be produced at a larger scale, whether production costs are compatible with commercial use, and which regulatory requirements must be met before they can be marketed as food ingredients. Third, dose–response and longer-term intervention studies are required to determine whether the acute vascular responses observed with watermelon rind are sustained during regular consumption and whether beetroot by-product formulations with defined nitrate content produce clinically relevant effects. These studies should establish realistic serving sizes, evaluate adherence and adverse events, and consider potential sources of variability, including oral hygiene practices, baseline endothelial function, medication use, and cardiometabolic health status. At present, the clinically relevant doses and long-term efficacy and safety of NO-targeted ingredients derived specifically from beetroot and watermelon by-products remain uncertain.

In conclusion, beetroot and watermelon by-products offer promising opportunities for functional food development. For beetroot peel and pomace, studies should measure nitrate content in the by-product ingredient and final food product and determine whether their consumption increases circulating nitrate and nitrite or improves vascular function. For watermelon rind, the preliminary findings should be confirmed in studies with more participants and repeated consumption over longer periods. Measurement of nitrate and L-citrulline content, reporting of the amounts consumed, and well-controlled human studies are necessary to determine the relevance of these by-products to cardiovascular health. 

## Data Availability

No datasets were generated or analysed during the current study.

## Key References

- Volino-Souza M, de Oliveira GV, Tavares AC, Souza K, Alvares TS: The effect of microencapsulated watermelon rind (Citrullus lanatus) and beetroot (Beta vulgaris L.) ingestion on ischemia/reperfusion-induced endothelial dysfunction: a randomised clinical trial. Food Funct 2023, 14(17):7959–7968.
  - ○ This randomized crossover trial showed that microencapsulated watermelon rind juice providing 4 g of L-citrulline attenuated the endothelial dysfunction induced by prolonged arterial occlusion in healthy adults. The study provides clinical evidence supporting watermelon rind as an NO–targeted functional ingredient.
- Volino-Souza M, de Oliveira GV, Conte-Junior CA, Alvares TS: Effect of microencapsulated watermelon (Citrullus lanatus) rind on flow-mediated dilation and tissue oxygen saturation of young adults. Eur J Clin Nutr 2023, 77(1):71–74.
  - ○ This study demonstrated that watermelon rind, a commonly discarded food by-product, improved endothelial function and increased circulating L-citrulline in healthy adults. The findings highlight the potential of watermelon rind as a sustainable nutritional strategy targeting NO bioavailability.
- Gorjanović S, Zlatanović S, Laličić-Petronijević J, Dodevska M, Micić D, Stevanović M, Pastor F: Enhancing composition and functionality of jelly candies through apple and beetroot pomace flour addition. NPJ Sci Food 2024, 8:85.
  - ○ This study showed that incorporation of beetroot pomace flour into jelly candies improved antioxidant activity and reduced postprandial glycemic responses while promoting food waste valorization. The work illustrates how agro-industrial by-products can be transformed into functional foods with potential cardiometabolic benefits.
